# High density lipoprotein mimicking nanoparticles for atherosclerosis

**DOI:** 10.1186/s40580-019-0214-1

**Published:** 2020-01-27

**Authors:** Jun Chen, Xixi Zhang, Reid Millican, Jacob Emil Creutzmann, Sean Martin, Ho-Wook Jun

**Affiliations:** Department of Biomedical Engineering, University of Alabama at Birmingham, Birmingham, AL USA

**Keywords:** Atherosclerosis, High-density lipoprotein, Nanoparticles, Therapeutic delivery, Imaging contrast agents, Theranostic agents

## Abstract

Atherosclerosis is a major contributor to many cardiovascular events, including myocardial infarction, ischemic stroke, and peripheral arterial disease, making it the leading cause of death worldwide. High-density lipoproteins (HDL), also known as “good cholesterol”, have been shown to demonstrate anti-atherosclerotic efficacy through the removal of cholesterol from foam cells in atherosclerotic plaques. Because of the excellent anti-atherosclerotic properties of HDL, in the past several years, there has been tremendous attention in designing HDL mimicking nanoparticles (NPs) of varying functions to image, target, and treat atherosclerosis. In this review, we are summarizing the recent progress in the development of HDL mimicking NPs and their applications for atherosclerosis.

## Introduction

Cardiovascular disease (CVD) is the leading cause of mortality and morbidity in the United States. It is reported that 24.3 million people were diagnosed with CVD, and 8 million deaths were reported to be associated with CVD in 2016 [1, 2]. The primary cause of CVD is atherosclerosis, which is a diseased state where the arteries become narrow and hardened due to the accumulation of plaque within the coronary arterial walls [3, 4]. Although great progresses have been made in the clinical diagnosis and treatment of atherosclerosis, the complexity of plaque pathophysiology poses significant challenges for effective diagnosis and treatment. Thus, persistent efforts have been made towards the development of highly effective approaches of specificity and selectivity for the diagnosis and therapy of atherosclerotic plaques.

Nanomedicine lies in the cutting-edge research field of nanotechnology in medicine that emphasizes on the design, fabrication, characterization, and application of nanoparticles (NPs) for the diagnosis, treatment and prevention for diseases [5]. NPs are nanostructures with controlled shapes and in nanoscale size, which have been applied for improving the treatment for a multitude of cancers clinically. NPs as platforms to deliver therapeutics to the cancers have been shown to provide several advantages. First, due to their nanoscale size, NPs will not induce risk of blocking vessels and can be administered through systemic administration. In addition, NPs have been reported to circulate longer than free drugs, which facilitate to prevent fast clearance from the body. Furthermore, NPs with a diameter less than 100 nm have been found to be taken up by endocytic vesicles. Moreover, the surface of NPs can be modified with ligands to target the disease sites to enhance the therapeutic efficacy of the anticancer drugs without causing significant cytotoxicity to normal tissues [6, 7]. Therefore, due to the observed promising properties of NPs in cancer therapy, many studies have been focusing on the development of NPs such as liposomes [8], polymeric NPs [9], and HDL mimicking NPs [9–15] for atherosclerosis treatment and imaging.

Among these NPs, HDL mimicking NPs have attracted tremendous attention for potential utility in atherosclerosis treatment and imaging, as HDL mimicking NPs are expected to possess similar structure and function to native HDL. Specifically, native HDL are dynamic NP systems of varied sizes ranging from 7 to 13 nm in diameter, which possess diverse shapes and compositions. Native HDL is mainly constituted of apolipoprotein A1 (apoA-I) and phospholipids, which  has been shown to be anti-inflammatory and anti-oxidative [16–18]. Significantly, native HDL was reported to possess great anti-atherosclerotic efficacy by interacting with ATP-binding cassette transporters A1 (ABCA1) and G1 (ABCG1) and the scavenger receptor B1 (SR-BI) in the plaque as cholesterol acceptors can remove the excess cholesterol from foam cells in atherosclerotic plaque via reverse cholesterol transport (RCT) [16–18]. In addition, native HDL were shown to prevent endothelial dysfunction by increasing the production of nitric oxide (NO) by activating endothelial nitric oxide synthase (eNOS) [19, 20]. Motivated by the excellent anti-atherosclerotic features of native HDL, the development of HDL mimicking NPs has escalated since its inception a decade ago and has made a great progress in this field thus far. Compared to other types of NPs which require drugs or targeting ligands to achieve therapeutic efficacy or targeting ability for atherosclerosis treatment and diagnosis, respectively, drug-free HDL mimicking NPs demonstrate intrinsic anti-atherosclerotic activity and targeting ability for atherosclerotic plaques. In addition, HDL mimicking NPs were also shown to have the ability to be encapsulated with drugs or diagnostic agents to increase the therapeutic efficacy or achieve the theranostic purpose for atherosclerosis.

Therefore, in the following sections, we will mainly summarize the recent progress in the development and utility of HDL mimicking NPs for atherosclerosis. We will first briefly introduce the methods to fabricate HDL mimicking NPs in Sect. 2. Then, we will discuss the recent progress of HDL mimicking NPs applied as therapeutic, imaging contrast, and theranostic agents for improving the efficacy for atherosclerosis diagnosis and treatment in Sect. 3. Lastly, we will provide critical future perspectives on the current advances of HDL mimicking NPs associated with atherosclerosis treatment and imaging in Sect. 4. This review will provide excellent up-to-date resources to help scientists in designing HDL mimicking NPs for atherosclerosis as well as to assist researchers who are interested in exploring the potential of HDL mimicking NPs for treating other diseases.

## Approaches for the fabrication of HDL mimicking NPs

The fabrication of HDL mimicking NPs is largely dependent on the amphipathic properties of apoA-I; its amphipathic characteristics allow for interactions with both the aqueous phase and the phospholipids, enabling apoA-I to stabilize the structure of small and two-layered discs of HDL mimicking NPs. After discoidal HDL mimicking NPs are loaded with the payloads, they can transform into spherical shaped NPs with a hydrophobic core of payloads coated with a phospholipid monolayer embedded with apoA-I. Thus, HDL mimicking NPs can exist as either discoidal or spherical in shape. With that in mind, the production of HDL mimicking NPs is mainly accomplished via two general approaches: reconstitution and microfluidic homogenization [21].

**Fig. 1:**
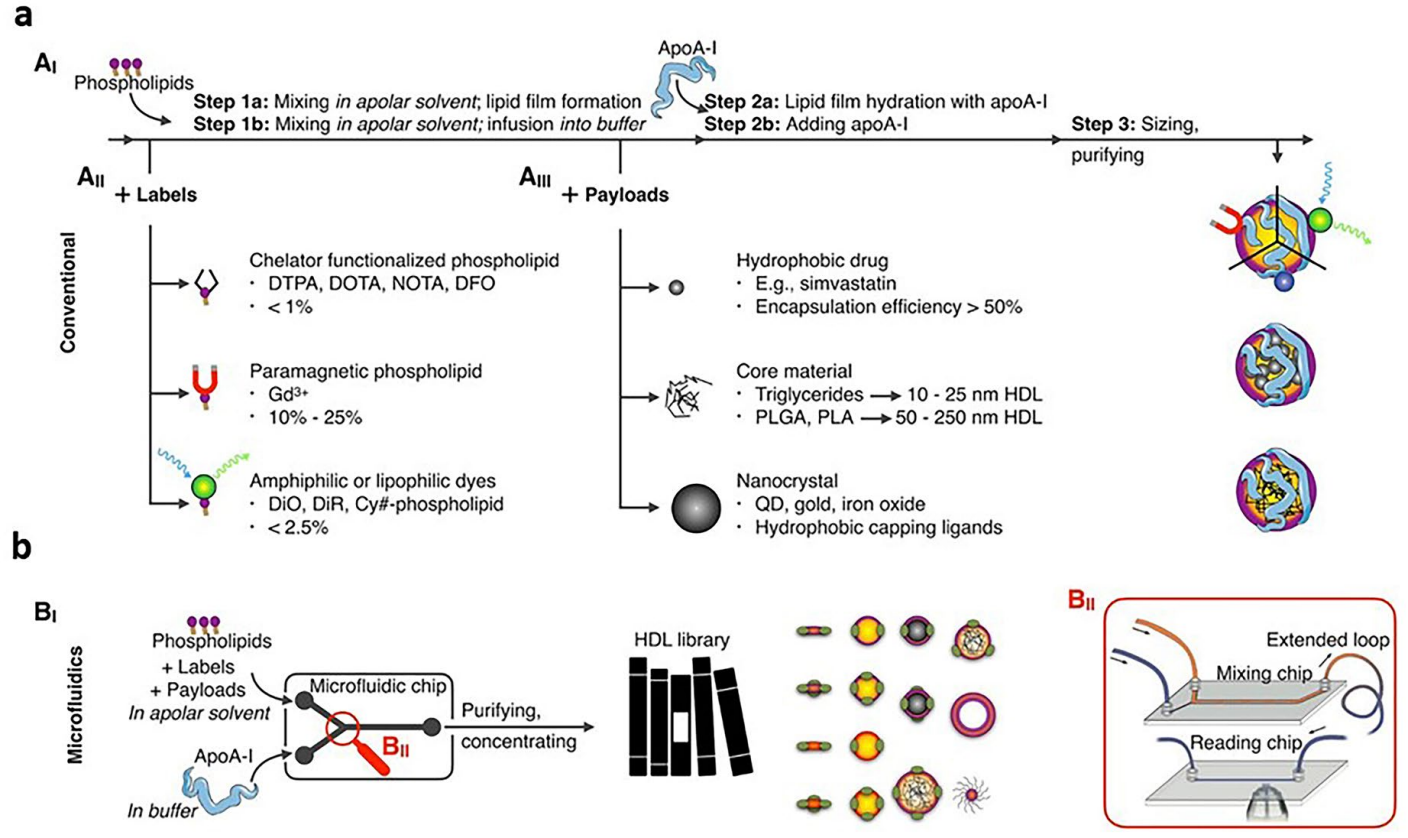
**a** HDL mimicking NP production. (A_I_) HDL mimicking NPs can be reconstituted per different “conventional” strategies and include (A_II_) amphiphilic labels or (A_III_) hydrophobic core payloads. **b** (B_I_) Microfluidic technology allows HDL mimicking NP instantaneous formation, (B_II_) a process that can be monitored by FRET confocal microscopy. Reproduce with permission [21]. Copyright 2018, American Chemical Society

Reconstitution is the traditional method of fabricating HDL mimicking NPs where HDL is reconstituted from apoA-I and phospholipids. The HDL mimicking NPs obtained by reconstitution are known as reconstituted HDL (rHDL) NPs. Desired features [21] such as labels (e.g. radioisotopes [22, 23], paramagnetic labels [24, 25], and fluorophore [22, 26, 27]), a diagnostically active nanocrystal core (e.g. iron oxide [28–30], quantum dot [31, 32], and gold [33–35]), and therapeutic payloads can be included in rHDL NPs for either imaging or targeted therapy during reconstitution (Fig. 1a) [21]. Particularly, rHDL NPs are assembled by first mixing phospholipids in an apolar solvent to form a lipid film, followed by hydrating the lipid film with apoA-I. They also can be formed by hydrating the lipids alone and then adding apoA-I [21]. The imaging labels of rHDL NPs are achieved by labeling the phospholipids with chelators such as gadolinium (Gd^3+^) [36, 37], and amphiphilic or lipophilic dyes [36] before mixing the lipids in an apolar solvent. The payloads are commonly encapsulated into rHDL NPs before adding apoA-I. The purification and size control of rHDL NPs can also be achieved via sonication [21].

In addition to traditional approach developed two decades ago, Mulder and his colleagues recently demonstrated the feasibility of using microfluidic devices to synthesize large scale of HDL mimicking NPs to address the limitations of traditional methods (Fig. 1b) [9, 21, 38]. By using this new approach, a continuous fabrication of HDL mimicking NPs of similar compositions and bioactivity to rHDL NPs can be obtained. The fabrication of HDL mimicking NPs begins with the formation of a phospholipid suspension and a hydrophobic payload in an apoA-I-containing buffer, followed by high-pressure homogenization through a microfluidizer high-shear fluid processor.

## Applications of HDL mimicking NPs for atherosclerosis

### HDL mimicking NPs as therapeutic delivery systems

As early as 2003, the anti-atherosclerotic efficacy of rHDL NPs was investigated in patients with acute coronary syndromes by the Tuzcu group. In this study, the authors demonstrated that rHDL NPs composed of ApoA-I Milano, a variant of apoA-I, were able to regress coronary atherosclerosis in patients [39]. Several following studies were also published regarding the investigation of the efficacy of other types of rHDL NPs for plaque regression in patients [40–42]. Because of the notable anti-atherosclerotic efficacy of rHDL NPs in patients, recently, researchers investigated the use of rHDL NPs as delivery systems to further improve the therapeutic efficacy of atherosclerosis treatment by taking advantage of the synergetic effect of therapeutics and rHDL NPs.

For instance, in 2014, Mulder and coworkers reported on the application of rHDL NPs to local delivery of statin (ST) to plaques to improve the bioavailability of the drug [10]. In this study, three types of rHDL NPs were fabricated: statin loaded rHDL NPs (ST-rHDL NPs), statin loaded rHDL NPs labeled with fluorescent dye (DiO-ST-rHDL NPs), and free rHDL NPs. By tracking DiO-ST-rHDL NPs, the authors noticed that these NPs could co-localize with CD68^+^ cells in the aortic root of ApoE^−/−^ mice. Furthermore, these NPs were found to be taken up more by macrophages than monocytes. Importantly, the authors also found that the three-month low dose (15 mg kg^−1^ statin, 10 mg kg^−1^ apoA-I) ST-rHDL NP infusion could inhibit the progression of inflammation, and the one-week high dose (60 mg kg^−1^ statin, 40 mg kg^−1^ apoA-I) ST-rHDL NP treatment could significantly decrease inflammation in ApoE^−/−^ mice. Soon later, a following study reported by the same group demonstrated that locally continuous inhibition of advanced plaque inflammation can be also achieved by 1-week high dose ST-rHDL NP administration (60 mg kg^−1^ statin, 40 mg kg^−1^ apoA-I, four infusions per week) followed by the eight-week oral ST treatment in ApoE^−/−^ mice [10].

Even though Mulder and coworkers demonstrated that ST-rHDL NPs exhibited good therapeutic efficacy for treating atherosclerosis, large amounts of drug loaded rHDL NPs may still accumulate in the liver due to the abundance of SR-BI in hepatocytes. Thus, Liu and coworkers investigated whether hyaluronic acid (HA) coating can decrease the undesired accumulation of rHDL NPs in liver but enhance the accumulation of NPs in the plaque [43]. The authors injected HA coated and non-coated rHDL NPs loaded with lovastatin (LT) in atherosclerotic New Zealand white (NZW) rabbits. The authors found that the number of HA-LT-rHDL NPs accumulated in the plaque was two times higher than that of the controls, the LT-rHDL NPs. In addition, the anti-protective efficacy of LT-rHDL NPs was also improved by HA coating, as smaller atherosclerotic lesion size with the least macrophage inflammation and matrix metalloproteinase expression was observed in HA-LT-rHDL NPs-treated atherosclerotic NZW rabbits compared to the control-treated rabbits. The increased accumulation of HA-LT-rHDL NPs may be due to the fact that HA coating can assist in preventing the rHDL NPs from binding with SR-BI in the liver and enhancing the targeting of CD44 in the injured endothelial cells (EC) in the plaques.

Despite the improvement in the local accumulation of rHDL NPs via HA coating, the lipid cores and the phospholipid monolayer of HA-LT-rHDL NPs may compromise the RCT function of rHDL NPs. To enhance the RCT pathway while simultaneously prevent macrophage targeting efficiency from getting compromised, in 2017, the same group developed an advanced HDL mimicking hybrid NP system composed of a poly(lactic-co-glycolic acid) (PLGA) core, a lipid bilayer structure, and conjugated HA [44]. The PLGA core was developed to encapsulate large amounts of the drug, achieve slow drug release, and maintain the spherical structure of rHDL NPs. On the other hand, the bilayer lipid shell was assembled to provide enough space and promote the removal of cholesterol from macrophages. In addition, the covalent conjugation of HA on the apoA-I of rHDL NPs provided a better shield for evading liver clearance [44]. By treating atherosclerotic NZW rabbits with HA-PLGA-rHDL NPs, the authors found that these NPs targeted macrophages in multiple stages: these NPs first traveled to the liver, then accumulated in the atherosclerotic plaque through the CD44-mediated pathway, and finally, were taken up by macrophages via SR-BI-mediated endocytosis [44]. Moreover, similar to their previous study, the enhancement of anti-atherosclerotic efficacy of the drug, simvastatin, by HA was also observed in atherosclerosis rabbit models [44]. In addition to HA, Liu and coworkers also demonstrated that dextran sulfate could promote the targeting efficiency and increase the anti-atherogenic efficacy of PLGA-rHDL NPs with atorvastatin (AT) to macrophages by promoting cholesterol efflux and decreasing macrophage oxidized low density lipoprotein (ox-LDL) uptake in vitro [45].

Next, to further maximize the anti-atherosclerotic efficacy of PLGA-rHDL NPs, in 2018, the same group developed novel dual-targeting HA-PLGA-rHDL NPs that can target macrophages and ECs sequentially via apoA-I and HA-modified dioleyl phosphatidylethanolamine (HA-DOPE). Significantly, the dual-targeting NPs loaded with two different therapeutic molecules (AT and lectin-like ox-LDL receptor-1 small interfering ribonucleic acid) showed an excellent ability to regress the atherosclerotic plaque. Moreover, the anti-atherosclerotic ability of HA-PLGA-rHDL NPs was shown to be enhanced as the HA molecular weight increased from an in vivo study using ApoE^−/−^ mice [46].

**Fig. 2:**
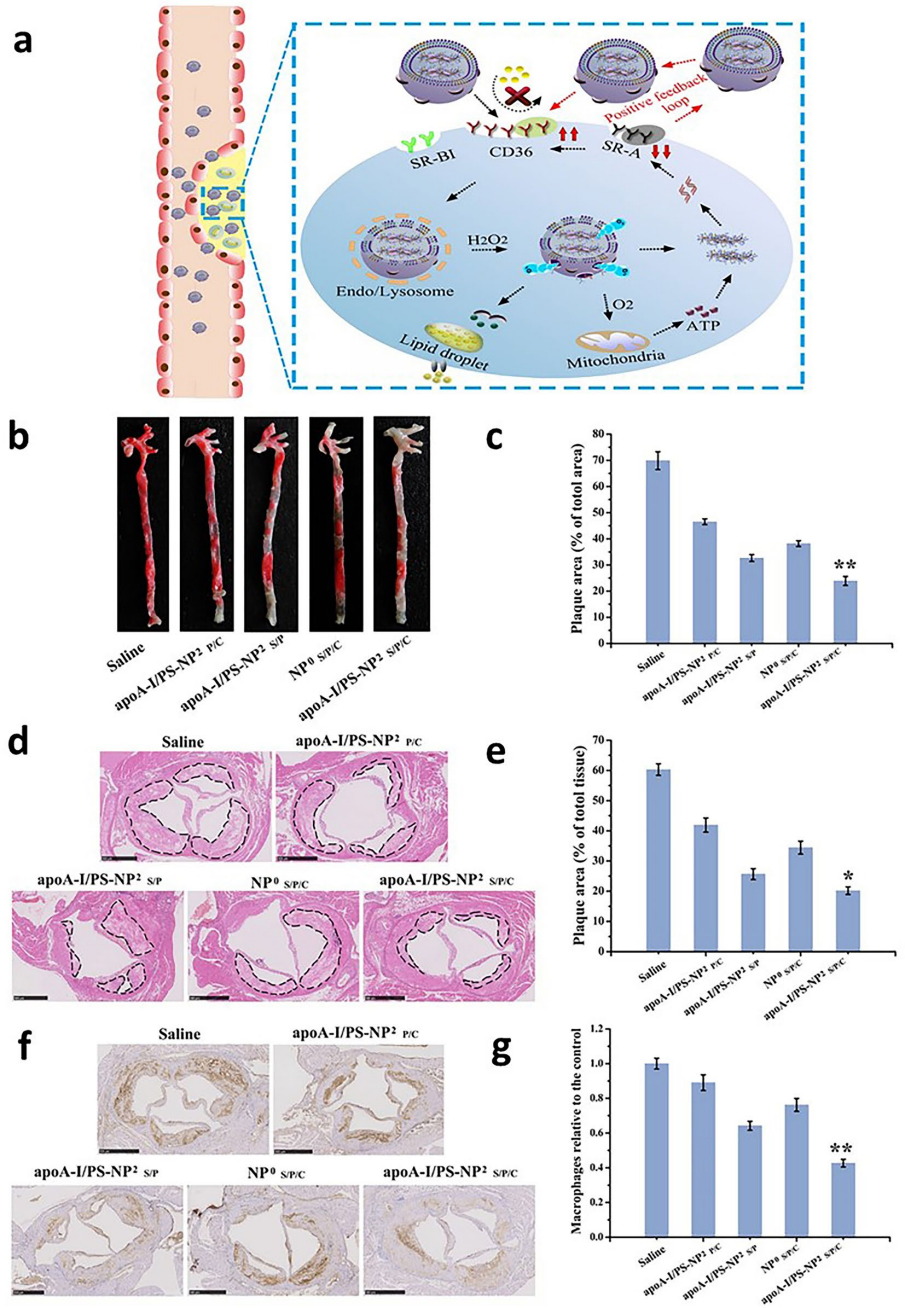
**a** Schematic illustration of dual-targeting biomimetic core–shell HDL mimicking NPs dynamically enhancing plaque targeting via a positive feedback loop and lowering intracellular lipid disposition. **b** Representative images and **c** quantitative data of aortic trees stained by oil red O. **d** Representative photographs and **e** quantitative analysis of plaque area based on H&E staining. **f** Representative images and **g** quantitative analysis of immunohistochemical staining of macrophages. Reproduce with permission [47]. Copyright 2019, Elsevier

In 2019, Liu and her coworkers designed another novel type of dual-targeting rHDL NPs to further enhance anti-atherosclerotic efficacy. The dual-targeting rHDL NPs were composed of an adenosine triphosphate (ATP)-responsive ternary polyplexes core, which  was loaded with scavenger receptor A (SR-A) targeting siRNA for upregulating the CD36 receptors and oxygen-evolving catalase for accelerating the ATP production for rapid release of SR-A siRNA [47]. The outer shells of the dual-targeting rHDL NPs were modified with apoA-I and phosphatidylserine to target SR-BI and the CD36 receptors of the macrophages as well as carry pitavastatin (PT) to remove cholesterol and inhibit the engulfment of LDL by macrophages (Fig. 2a). In vivo evaluation of the NPs in ApoE^−/−^ mouse models demonstrated that, when compared to the control, these NPs showed 3.3-fold higher of accumulation in the plaque, therefore leading to a 65.8% plaque reduction, which is relatively significant anti-atherosclerotic activity among rHDL NPs (Fig. 2b–g). In the same year, another study conducted by Liu and coworkers reported on the development of LT loaded spherical rHDL NPs, LT-s-rHDL NPs, and the effect of the LT:s-rHDL ratio on its anti-atherosclerotic effect. The authors showed that the NPs with the ratio of LT:s-rHDL of approximately 10 had the best synergistic effect in inhibiting ox-LDL uptake by macrophages in vitro and were able to reduce 24.9% of the plaque area in vivo compared to the control [47]. Similarly, in 2018, Chen and coworkers investigated the effect of monosialoganglioside (GM1) on the anti-atherogenic efficacy of LT-rHDL NPs. The authors observed that the LT-GM1-rHDL NPs exhibited more effectiveness in terms of sustained release of LT, blood circulation, plaque targeting, accumulation in liver, and stronger anti-atherogenic activity (~ 37% plaque reduction) compared to rHDL NPs without GM1 (~ 29%) in vitro [48].

Cluster of differentiation 40 (CD40) is a well-known protein receptor that can recruit tumor necrosis factor receptor-associated factors (TRAFs) to drive atherosclerosis. However, long-term suppression of CD40 leads to immune suppression and thrombosis. Therefore, Seijkens and coworkers studied an rHDL NP-mediated approach to suppress atherosclerosis by only targeting the interaction between TRAF6 and CD40 using a small molecule inhibitor (687702). This approach allowed to maintain the interaction between TRAF2/3/5 and CD40 (CD40-mediated immunity). The in vivo study demonstrated that TRAF6-rHDL NPs could reduce the expression of CD40 and integrin in classical monocytes, thereby reducing monocyte recruitment and impeding the initiation of atherosclerosis. More importantly, TRAF6-rHDL NPs displayed therapeutic efficacy for treating established atherosclerosis by halting plaque progression and stabilizing the plaque. This was demonstrated by the significant decrease of plaque size in rHDL-TRAF6 NP-treated ApoE^−/−^ mice for 6 weeks [49].

To gain a more comprehensive evaluation of the TRAF6-rHDL NPs, in 2018, the efficacy and safety of TRAF6-rHDL NPs were assessed in atherosclerotic mice and non-human primates by the Duivenvoorden, Lutgens and Mulder groups. Particularly, 1 week of four TRAF6-rHDL NPs infusions was found to regress atherosclerotic plaque in mice. Although the biodistribution study demonstrated that only 1.3% of TRAF6-rHDL NPs accumulated in the aorta, 86% and 81% of monocytes in the aorta were found to taken up TRAF6-rHDL NPs. Moreover, 67% and 65% less macrophages and T cell contents were observed in TRAF6-rHDL NP-treated mice compared to the untreated ones, respectively. The anti-atherosclerotic efficacy of TRAF6-rHDL NPs was due to their ability to impair the migration capacity of monocytes and decrease monocyte recruitment by downregulating the genes involved in regulating monocyte migration but upregulating the genes associated with lymphocyte homing and cell adhesion for athero-protective function. For the non-human primate model, large amounts of TRAF6-rHDL NPs were found to accumulate in the liver and kidneys 72 h after infusions [50].

Recently, efforts also have been made to develop apoA-I mimetic peptides to fabricate HDL mimicking NPs to lower the cost and shorten the time required for extracting apoA-I. For instance, in 2013, Ghadiri and coworkers assembled HDL mimicking NPs by using synthetic apoA-I mimetic peptides of 23 or 16 amino acids, and found that HDL mimicking NPs assembled by multimeric and multivalent peptides demonstrated better function in cholesterol efflux compared to the monomeric counterpart [51]. Later in 2014, Ghadiri and colleagues evaluated the in vivo efficacy of HDL mimicking NP containing apoA-I mimetic trimers in Ldlr^−/−^ mouse models. It was surprising to observe that these HDL mimicking NPs containing apoA-I mimetic trimers reduced 50% of the atherosclerotic lesion via oral administration [52]. Likewise, in 2018, Chen and coworkers reported an advanced approach to upregulate ABC transporters and increase cholesterol receptors by using HDL mimicking NPs fabricated with a 22-amino acid apoA-I-mimetic peptide (22A). In this study, T1317, an activation agonist for liver X receptor (LXR), was delivered to atherosclerosis plaque to activate ABCA1 using HDL mimicking NPs fabricated by homogenization. They found that T1317-HDL mimicking NPs neither induced serum triglyceride level increase nor upregulation of LXR-target gene expression in the liver but reduced 40.8% area of plaque formation in mouse models compared to the control group. The therapeutic efficacy of the T1317-HDL mimicking NP resulted from the fact that HDL mimicking NPs served as cholesterol acceptors and LXR could induce cholesterol efflux [53].

In addition, to optimize the anti-atherosclerotic efficacy of HDL mimicking NPs loaded with therapeutic compounds developed by microfluidic homogenization, in 2016, Mulder and coworkers created a library of LXR agonist (GW3965) loaded hybrid HDL NPs (GW3965-HDL mimicking NPs) with different sizes (10 nm, 30 nm, and over 100 nm), shapes (discoidal or spherical shape), and compositions (different phospholipids—PLGA or PLA) via either reconstitution or microfluidic homogenization. The authors reported that the cargo-free HDL mimicking NPs made with 1-palmitoyl-2-oleoyl-sn-glycero-3-phosphocholine (POPC) demonstrated the best efficiency for promoting cholesterol efflux, while the polymer core HDL mimicking NPs showed the least. This observation may be due to the fact that the polymer cores were so rigid that they may decrease the flexibility of apoA-I, which was in great contrast to the POPC core HDL mimicking NPs that were much flexible. Also, the authors found that smaller HDL mimicking NPs had longer blood half-lives and the HDL mimicking NPs with the size of 30 nm showed the longest half-life. The targeting ability of these NPs were also different. After a systematic study, the authors found that 30 nm GW3965-HDL mimicking NPs composed of POPC were the best candidates as they demonstrated a long half-life, high efficiency for cholesterol efflux, and good accumulation in the macrophages in the plaque [54].

**Fig. 3:**
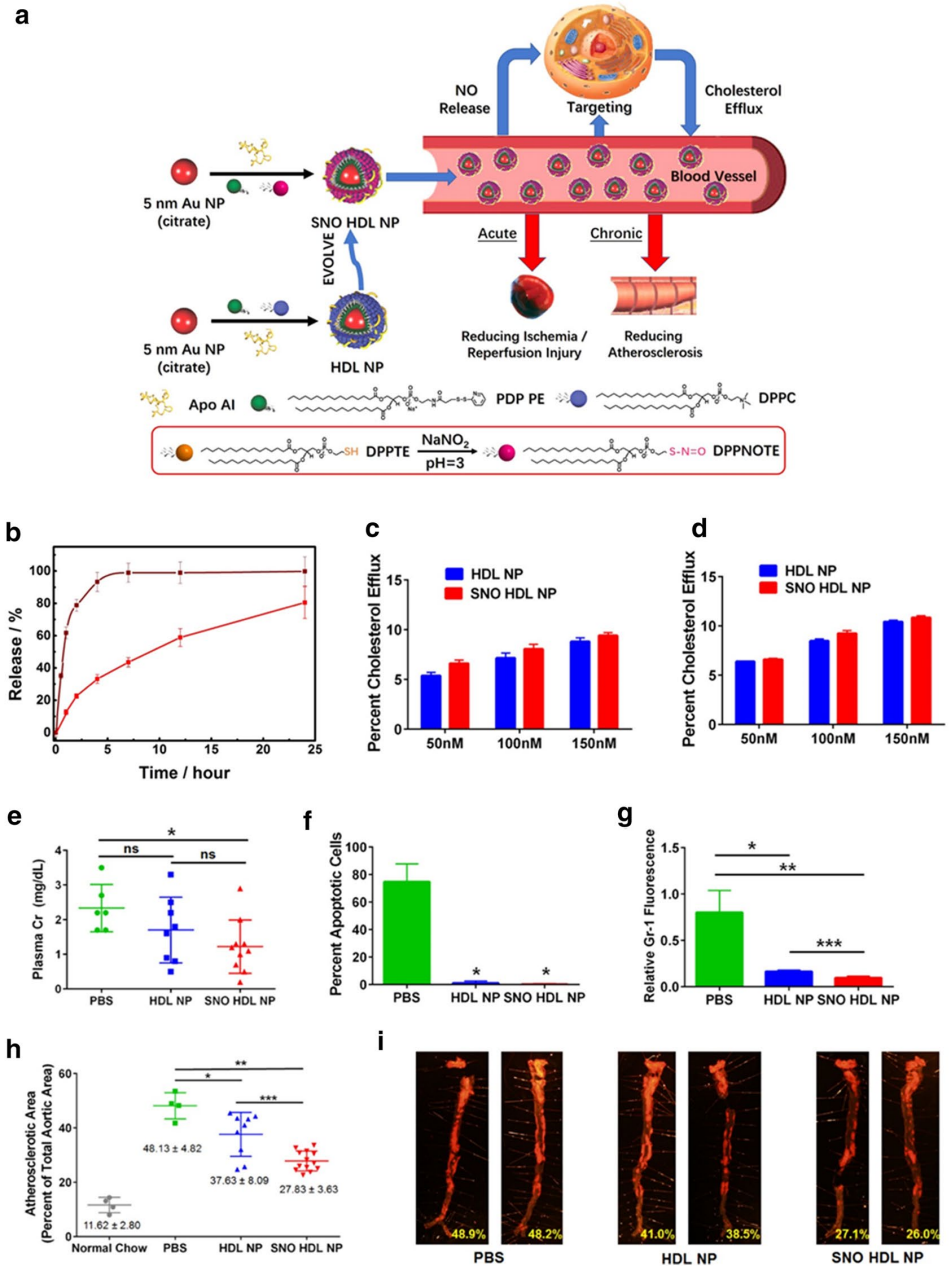
**a** Preparation, properties, and functions of SNO-HDL mimicking NPs. **b** NO release from SNO-HDL mimicking NPs (red solid line) and DPPNOTE (brown solid line) at 37 °C. **c**, **d** Cholesterol efflux from 3H-cholesterol loaded J774 macrophages (**c**) and differentiated THP-1 macrophages (**d**) to SNO-HDL mimicking NPs. **e** Plasma creatinine levels of mouse kidney transplant recipients on day 2 post transplantation. **f** Quantification of apoptosis staining in kidney transplant recipients. **g** Quantification of immunocytochemistry staining for Gr-1, a neutrophil marker, in kidney transplant recipients. **h** Comparison of atherosclerotic area of ApoE^−/−^ mice fed with a high-fat diet (HFD) for 12 weeks, then administered with PBS, HDL mimicking NPs, or SNO-HDL mimicking NPs for an additional 6 weeks. **i** Representative images of aortas from mice treated with PBS, HDL mimicking NPs, or SNO-HDL mimicking NPs. Red fluorescent staining indicates atherosclerotic plaques. Reproduce with permission [55]. Copyright 2018, American Chemical Society

Moreover, an interesting study reported by the Thaxton group showed that NO-releasing HDL mimicking NPs (SNO-HDL NPs) showed great efficacy for preventing atherosclerosis progression. The SNO-HDL NPs were fabricated by the incorporation of *S*-nitrosylated phospholipid (DPPNOTE), a NO donor, into HDL mimicking NPs composed of a gold core, apoA-I, and lipids (Fig. 3a). In vitro characterization of SNO-HDL NPs showed that these NPs did not induce toxicity to human aortic endothelial cells (hAECs), human aortic smooth muscle cells (hAoSMCs), and human monocytes (THP-1). An in vitro NO release study demonstrated that SNO-HDL NPs showed an initial rapid release of NO in the first 2 h, followed by a decreased NO release in 2–7 h, and finally an even lower release of NO in 7–24 h with the total release of 80.53 ± 9.90% of NO at 24 h, which is in great contrast to the free DPPNOTE that showed a burst release of NO, 93.29 ± 5.88% at 4 h (Fig. 3b). Furthermore, the authors demonstrated that SNO-HDL NPs possessed a similar ability to native HDL for cholesterol efflux from J774 macrophages and differentiated macrophages from THP-1 monocytes (Fig. 3c, d). In addition, SNO-HDL NPs demonstrated a better efficiency for inhibiting the migration of hAoSMCs than free DPPNOTE in vitro. Lastly, the in vivo study demonstrated that the mice only developed 27.83% of atherosclerotic plaque after treated with SNO HDL-NPs, which was much less than that of the PBS-treated mice (48.13 ± 4.82%) (Fig. 3e–i) [55].

Besides NO, few groups studied to use HDL mimicking NPs to deliver biomolecules. For instance, in 2010, Thaxton and coworkers investigated to use HDL mimicking NPs to deliver antisense cholesterylated DNA to cells. The in vitro study demonstrated that the DNA-HDL mimicking NPs were able to knockdown miR-210, decrease the expression of E2F3a and increase STAT1 expression [35]. Later in 2017, Liu and coworkers developed HDL mimicking NPs condensed with polyethyleneimine (PEI) for the delivery of anti-microRNA155, the complementary sequence against microRNA155 (miR-155), for treating atherosclerosis. MiR-155 was reported to promote foam cell formation, which, in turn, causes it to be expressed in the macrophages in the plaque. The in vitro study demonstrated that anti-miR-155-HDL/PEI NPs were able to target macrophages, avoid endocytosis, and exhibit high transfection efficacy. However, the evaluation of anti-miR-155-HDL/PEI NPs in vivo was not conducted in the current study [56]. Recently in 2019, Thaxton and coworkers developed DNA-PL4 HDL hybrid NPs. The DNA-PL4 HDL hybrid NPs were synthesized by first preparing the azide-phospholipid conjugate (PL4) core scaffold lined with 9-mer dsDNA (9-DNA-PL4) or 18-mer dsDNA (18-DNA-PL4), followed by adding dipalmitoylphosphatidylcholine (DPPC) liposomes and apoA-I to the scaffold. Both PL4 HDL and DNA-PL4 HDL hybrid NPs developed were found to closely mimic the secondary structure of native HDLs and show a great ability of facilitating cholesterol efflux from cholesterol loaded J774 macrophages. More importantly, when macrophages were treated with PL4 HDL and DNA-PL4 HDL hybrid NPs, NF-κB activity—the hallmark of cardiovascular disease—was decreased by 31% and 16%, respectively. However, no in vivo evaluation of these DNA-PL4 HDL hybrid NPs was shown in this study [57].

### HDL mimicking NPs as imaging contrast and theranostic agents

#### HDL mimicking NPs as imaging contrast agents

From the early 2000s to the present day, HDL mimicking NPs are studied for atherosclerosis imaging and diagnosis [24, 58]. However, there are not as many recent studies that investigated the use of HDL as imaging contrast agents as those related to the therapeutic function of HDL. Thus, in this section, we are mainly highlighting works that emphasize the potency of HDL mimicking NPs as imaging contrast agents.

In 2004, Fayad and colleagues developed gadolinium-based contrast agents HDL (GBCA-HDL) mimicking NPs using apoA-I from human plasma, gadolinium diethylenetriaminepentaacetic acid- 1,2-dimyristoyl-sn-glycero-3-phosphoethanolamine (Gd-DTPA-DMPE), and phospholipids for magnetic resonance imaging (MRI). The developed GBCA-HDL mimicking NPs showed a value of T1 relaxation rate, r1 = 10.4 mM^−1^ S^−1^, in water and predominantly localized in the macrophages of the atherosclerotic plaque within the aorta of ApoE^−/−^ mice with maximum MRI contrast intensity 24 h post-injection. Although the use of GBCA-HDL mimicking NPs showed promising results within this study, further advancements in imaging technology was required to increase imaging capability and specificity to atherosclerosis plaques [59]. Due to the concern regarding safety of using apoA-I from human plasma, in 2008, Favad and coworkers developed a novel type of GBCA-HDL mimicking NPs using 37 pA, an apoA-I mimetic peptide, which was able to perform cholesterol efflux. These types of GBCA-HDL mimicking NPs also showed in vivo efficacy in ApoE^−/−^ mouse models [24]. Additionally, later in 2013, the same group conducted a study for clarifying the mechanism of using the Gd-HDL mimicking NPs for delivering Gd to the plaque and their fate in plasma [60]. In vitro and in vivo studies indicated that the possible delivery mechanism was that the Gd-DTPA-DMPE could be spontaneously transferred to the endogenous phospholipids and then cleared from the plasma [60].

To further improve the contrast intensity in vivo, several groups were working on improving the imaging effectiveness of GBCA-HDL mimicking NPs in the plaque. For instance, in 2008, Fayad and colleagues advanced the GBCA-HDL mimicking NPs imaging efficacy by modifying the NPs with P2A2, a lipopeptide derived from apolipoprotein E, which was expected to assist the NPs in penetrating cell membranes and thus promote the uptake of NPs into the cells. The in vivo study demonstrated that the P2A2-HDL mimicking NPs showed pronounced MRI signal enhancement than the NPs without P2A2 incorporation. Moreover, those P2A2 modified NPs showed higher normalized enhancement ratio (NER) (90%) than that of the unmodified NPs (53%) after 24 h injection [15].

In 2014, Sigalov and coworkers developed a novel type of macrophage targeting GBCA-HDL mimicking NPs by replacing the native apoA-I with either oxidative apoA-I or oxidized synthetic 22-mer peptides similar to amphipathic helices 4 (H4) and 6 (H6) of apoA-I for the fabrication of the NPs. An in vitro study demonstrated that a significantly larger amount of GBCA-HDL mimicking NPs composed of oxidized apoA-I or peptides were taken up by the macrophages than their unmodified counterpart. Moreover, a significant enhancement of MRI signal was observed by using GBCA-HDL mimicking NPs fabricated with oxidized peptides (normalized enhancement ratio (NER):120%) instead of NPs composed of unmodified apoA-I (NER:45%) in ApoE^−/−^ mice [61].

**Fig. 4:**
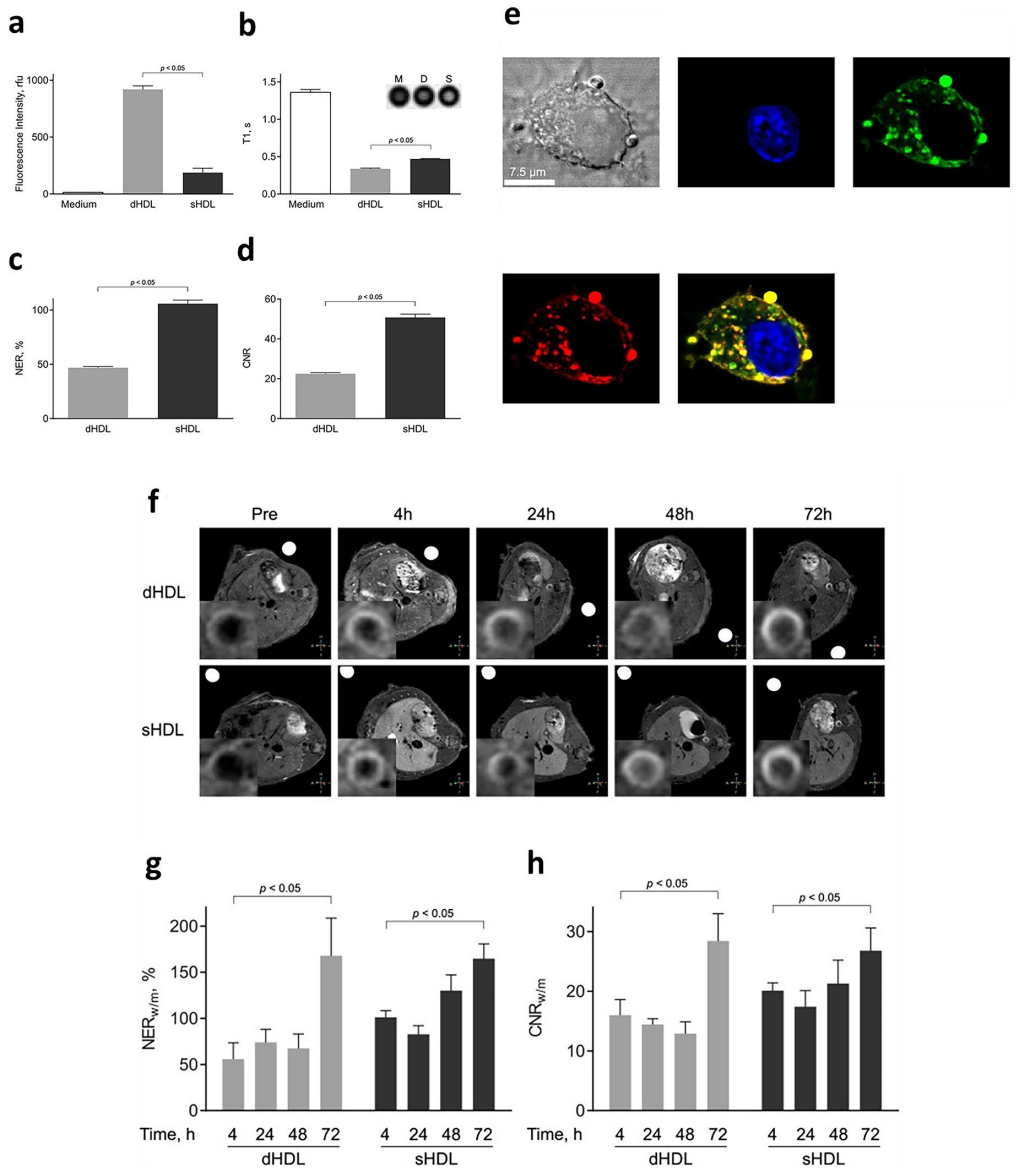
774A.1 macrophages were incubated for 2 h at 37 °C with medium only or with medium containing 2.0 μM paramagnetic and Rhodamine B-labeled discoidal HDL (dHDL) or spherical HDL mimicking NPs synthesized using a 1:1 mixture of oxidized synthetic apoA-I peptides H4 and H6. **a** Fluorescence intensities of cell lysates were measured and normalized to total cell protein content. **b** Loosely packed cell pellets were generated and T1 values were measured using T1-weighted MR imaging of cell pellets incubated with medium alone, dHDL mimicking NPs or sHDL mimicking NPs. **c** Normalized enhancement ratio (NER) values for cell pellets are calculated from corresponding T1-weighted images and are relative to cells incubated with medium only. **d** Contrast-to-noise ratio (CNR) values for cell pellets are calculated from the corresponding T1-weighted images and are relative to cells incubated with medium only. **e** Differential interference contrast (DIC) image of a single representative J774A.1 macrophage incubated with sHDL (similar images were obtained for dHDL). 4′,6-diamino-2-phenylindole (DAPI) dye (blue), Dylight 488-labeled peptide H4 (green), Rhodamine B-labeled lipid (red) and merged image. **f** Representative axial T1-weighted images of ApoE^−/−^ mice, collected before (Pre) and 4 h, 24 h, 48 h and 72 h after administered with the equivalent of 0.05 mmol Gd kg^−1^ of paramagnetic and Rhodamine B-labeled dHDL or sHDL mimicking NPs. The insets in each scan show the original images, which were cropped and enlarged to highlight the aorta. **g** NER values (mean ± SD) of mouse aorta wall following contrast administration were calculated for each of the time points (n = 5 slices) for the representative mouse using the corresponding T1-weighted images and are relative to muscle. **h** CNR values (mean ± SD) of mouse aorta wall following contrast administration were calculated for each of the time points (n = 5 slices) using the corresponding T1-weighted images and are relative to muscle. Reproduce with permission [62]. Copyright 2015, Public Library of Science

Later in 2015, to further increase the imaging effectiveness of macrophage targeting GBCA-HDL mimicking NPs, in a following study, Sigalov and coworkers increased the GBCA content for GBCA-HDL mimicking NPs and achieved a significant contrast enhancement NER value using these NPs (NER > 165%). More importantly, the authors also studied the shape effect (spherical versus discoidal shape) on fluorescence intensity, T1 value and NER value of GBCA-HDL mimicking NPs in vitro, and found that discoidal GBCA-HDL mimicking NPs had a higher fluorescence intensity but lower NER and contrast-to-noise (CNR) values compared to spherical NPs (Fig. 4a–d). They also showed that both types of GBCA-HDL mimicking NPs can be engulfed by macrophages **(**Fig. 4e). Additionally, good targeting and imaging abilities of both types of GBCA-HDL mimicking NPs were observed in vivo by MRI (Fig. 4f, g) [62]. Chen and colleagues also reported the improvement of the efficacy of GBCA-HDL mimicking NPs for MRI by targeting collagen in the plaque by incorporating collagen-specific EP3533 or EP3612 peptides into GBCA-HDL mimicking NPs. Their study demonstrated the feasiblity to monitor plaque progression by collagen amounts via the intensity of the MR signal from GBCA-HDL mimicking NPs [13].

Besides GBCA-HDL mimicking NPs, inorganic nanocrystal core HDL mimicking NPs were also developed for imaging. In 2008, Mulder and colleagues reported the development of gold (Au), iron (II) oxide (FeO) and quantum dots (QD) encapsulated HDL mimicking NPs for either computer tomography (CT) or MRI using conventional multistep methods. The authors showed that the Au-HDL mimicking NPs demonstrated contrast in both imaging modalities, while FeO- and QD-HDL mimicking NPs provided T2-weighted and T1-weighted MRI contrast, respectively. Additionally, an in vivo study showed that the NER between pre-scan and post-scan of Au-, QD-, and FeO-HDL mimicking NPs were 139 ± 25%, 69 ± 23% and 53 ± 11%, respectively [33].

Later in 2011, the same group studied the biological characteristics of FeO-HDL mimicking NPs in vitro and in vivo. The authors demonstrated that FeO-HDL mimicking NPs can be taken up by macrophages individually and were stable in circulation. Moreover, the authors also found that the cholesterol efflux capacity of FeO-HDL mimicking NPs was very similar to that of native HDL in vitro [28]. Similar to FeO-HDL mimicking NPs, Au-HDL mimicking NPs were also shown to possess similar cholesterol efflux capabilities to that of native HDL shown by Thaxton and colleagues [34]. Moreover, in 2013, Mulder and coworkers demonstrated successful fabrication of Au-, QD-, and FeO-HDL mimicking NPs that can show contrast in both MRI and CT imaging by using the single-step approach via microfluidic devices [38].

In 2014, instead of using FeO, Au and QD, Jung and coworkers developed HDL mimicking NPs with superparamagnetic iron oxide nanoparticles (SPIOs) or ^59^Fe-SPIOs. The blood clearance, biodistribution, and ability of detection of atherosclerosis lesion of these NPs in ApoE^−/−^ mice after intravenous (IV) and intraperitoneal (IP) injection were also characterized [29]. Particularly, in vitro characterization of these NPs indicated similar cholesterol and triglyceride fraction of isolated HDL from human plasma. The blood clearance test exhibited a biexponential decrease for IV and slow increase for IP but both administrative approaches achieved similar steady states after 40 min [29]. For organ biodistribution, both approaches showed predominant accumulation in the liver and the spleen, while IP had significantly more accumulation in the aorta. Moreover, the increased uptake after IP administration was also assessed via ex vivo fluorescence microscopy for peritoneal macrophages, MRI and histological staining for atherosclerotic plaque, and X-ray fluorescent maps for the aorta [29]. Therefore, the authors demonstrated that IP injection might achieve higher uptake in vessel wall lesions, and thus, making it one of the optimal approaches in imaging atherosclerotic plaque using HDL mimicking NP labeled with inorganic NPs as well as in multimodal imaging [29].

Besides their utility for MRI and CT  imaging, HDL mimicking NPs can be also modified to show positron emission tomography (PET) signals. In 2016, Mulder and coworkers developed and evaluated two radiolabeled HDL NPs to visualize HDL mimicking NP behavior in vivo using PET noninvasively [63]. The HDL mimicking NPs were prepared via reconstitution of apoA-I or phospholipid (1,2-dimyristoyl-sn-glycero-3-phosphocholine), then radiolabeled by zirconium-89 (^89^Zr) to either apoA-I and phospholipid to form ^89^Zr-A1-HDL mimicking NPs and ^89^Zr-PL-HDL mimicking NPs, respectively [63]. The biodistribution and lesion targeting capability of these NPs were evaluated in murine, rabbit and porcine atherosclerosis models. The authors found that both HDL mimicking NPs mainly accumulated in the kidney, liver and spleen—specifically, ^89^Zr-A1-HDL demonstrated very high kidney uptake while ^89^Zr-PL-HDL showed a relatively even distribution [63]. Moreover, there were significantly more of both HDL NPs observed in the atherosclerotic aorta than the control, indicating their preferential targeting ability to plaques. In short, ^89^Zr labeled HDL NPs allowed for the observation of HDL behavior in vivo and the different labeling strategies provided the investigation of respective HDL components (^89^Zr-A1-HDL for apolipoproteins and ^89^Zr-PL-HDL for complex lipid exchange process) [63].

#### HDL mimicking NPs as theranostic agents

HDL mimicking NPs were also investigated as theranostic agents for atherosclerosis imaging and treatment. For instance, in 2013, fluorescent HDL mimicking NPs (TPP-HDL-apoA-I-quantum dots (QD) NPs) were fabricated by Dhar and coworkers using PLGA, PLGA-b-PEG-QD, cholesteryl oleate, stearyl-TPP, DSPE-PEG-COOH, and the apoA-I mimetic 4F peptide. PLGA and cholesteryl oleate were the main components of the NP core. The PLGA-b-PEG-QD was used for inducing fluorescence during optical imaging. The apoA-I mimetic 4F peptide and DSPE-PEG-COOH were coated outside the core, which were utilized for mimicking the structure of HDL NPs. Stearyl-TPP is a ligand that can target the collapse of the mitochondrial membrane potential, which was used for evaluating apoptotic activity of cells in the plaque. The developed HDL mimicking NPs showed great cholesterol binding properties and an excellent capability of detecting cell apoptosis [11].

**Fig. 5:**
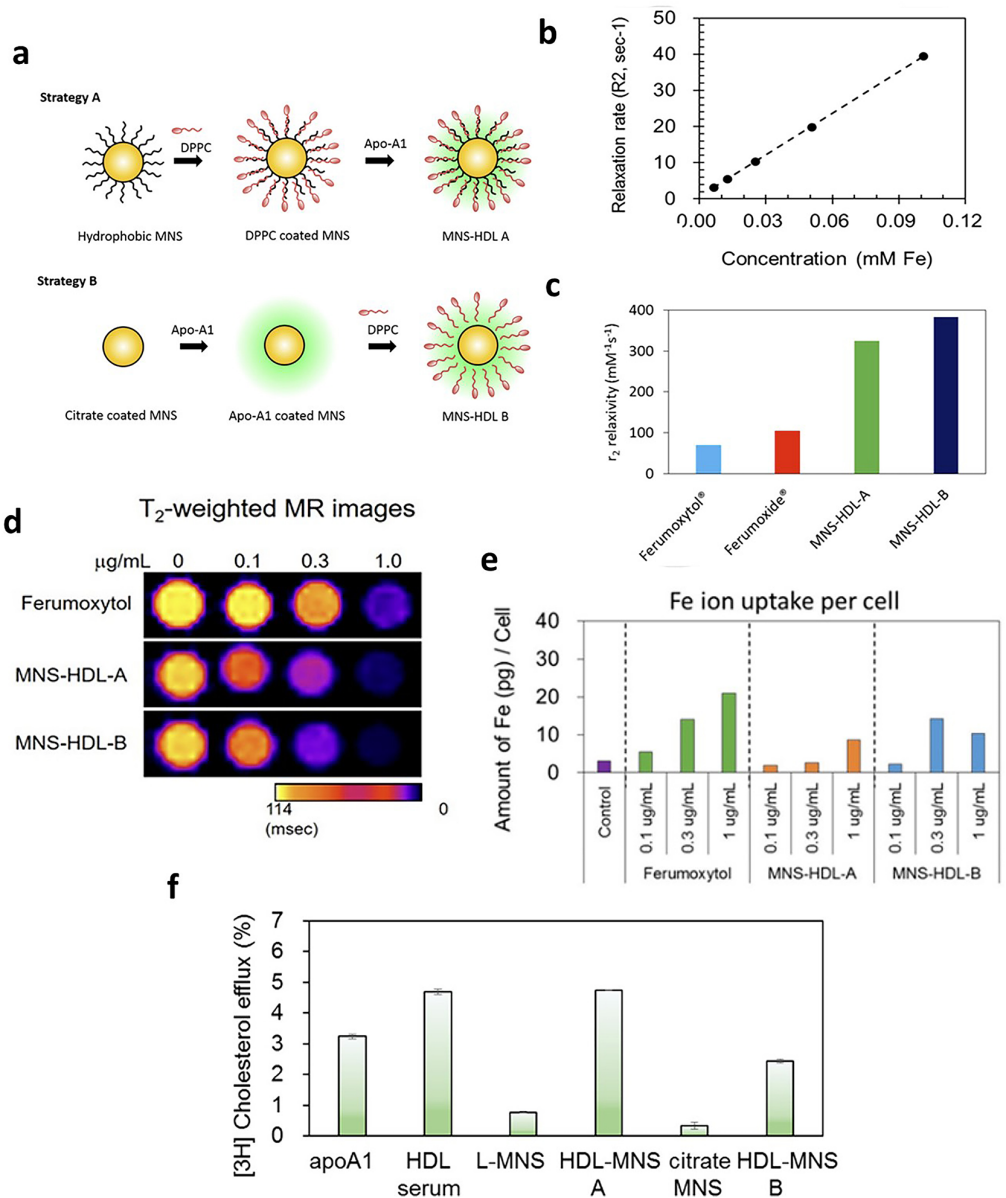
**a** Approach 1 to synthesize HDL-MNS A particles where oleic acid coated hydrophobic MNS were first coated with a neutral lipid 1,2-dipalmitoyl-sn-glycero-3-phosphocholine (DPPC) and later coated with apolipoprotein A1 (apoA-1). In approach 2, hydrophilic MNS were first coated with apoA1 and then coated with DPPC, resulting in HDL-MNS B particles. **b** *r*_2_ relaxivity plot of HDL-MNS B particles measured at 1.4 T. **c** Comparison of *r*_2_ values of HDL-MNS A and B with commercially available contrast agents (ferumoxytol, and ferumoxide). **d** T2-weighted MR images shows darker signal (decrease in T2 relaxation time) with the increase of Fe concentration. **e** Amount of Fe ion taken up per cell was measured via ICP-MS of cell pellets fed with difference concentrations of Ferumoxytol and HDL-MNS **f** cholesterol efflux from J774 macrophage cell lines by HDL-MNS. Reproduce with permission [64]. Copyright 2017, American Chemical Society

Later in 2017, the Dravid group developed biocompatible theranostic HDL mimicking magnetic nanostructures (HDL-MNS) by coating HDL on the magnetic iron (II, III) oxide (Fe_3_O_4_) NPs, which was expected to localize and target macrophages by MRI and induce cholesterol efflux from macrophages. In the study, hydrophobic and hydrophilic Fe_3_O_4_ NPs were used to generate HDL-MNS A and HDL-MNS B with r_2_ relaxivity values of 340 and 382 mM^−1^ S^−1^, respectively. The r_2_ relaxivity value of HDL-MNS is ~ 5 times higher than that of Ferumoxytol, a T2 contrast agent used in clinical patients. The higher value may result from the aggregation of MNS in the HDL-MNS, which could lead to a lower use amount of HDL-MNS to achieve the same contrast as Ferumoxytol (Fig. 5a–c). A similar result was also observed in the in vitro MRI of HDL-MNS and Ferumoxytol in J77R macrophages. When the same iron concentration was used, the HDL-MNS demonstrated higher T2 relaxation time than Ferumoxytol, indicating a better contrast enhancement of HDL-MNS than Ferumoxytol (Fig. 5d, e). Moreover, the authors also investigated the therapeutic function of HDL-MNS by measuring the NP-induced cholesterol efflux from macrophages fed with lipids. The respective cholesterol efflux percentage of HDL-MNS A was 4.8%, which was better than HDL-MNS B (2.4%) and apoA-I alone (3.2%), and similar to that of native HDL (4.7%) (Fig. 5f) [64].

In a recent study in 2019, Mulder and coworkers developed a strategy for the large-scale production of nano-immunotherapeutic ST-HDL mimicking NPs for a large animal study. The authors designed a method combining the large-scale extraction of apoA-I and the production of ST-HDL mimicking NPs using apoA-I and phospholipids through high-pressure microfluidic homogenization. This approach was able to produce 18 g of ST-HDL mimicking NPs with good morphology per run with an excellent production rate that was 80-fold higher than that of previous HDL mimicking NPs fabrication methods. In order to quantitatively evaluate the half-life and biodistribution as well as the therapeutic efficacy of ST-HDL mimikcing NPs in multiple animal models using PET, the authors labeled the ST-HDL mimicking NPs with zirconium-89 (^89^Zr). By using ApoE^−/−^ mouse models, the authors found that the half-life of the [^89^Zr]-ST-HDL mimicking NPs was around 3.1 h and observed a high accumulation of these NPs in atherosclerotic plaque. Within 1 week of [^89^Zr]-ST-HDL mimicking NPs infusion (60 mg kg^−1^ ST per infusion), the number of macrophages in the plaque decreased by 45%, and neither liver toxicity nor metabolic changes were observed. Moreover, the assessment of biodistribution of these NPs was also evaluated using rabbit and pig atherosclerosis models by PET/MRI. The authors demonstrated that the [^89^Zr]-ST-HDL mimicking NP infusion neither induced significant inflammation in animal vessels nor affected the proliferation of cells and blood cholesterol concentration in pig atherosclerosis models; however, a reduction of vessel wall area by [^89^Zr]-ST-HDL mimicking NP infusion was observed in rabbit atherosclerosis models. Based on the data, although there is a great potential of using [^89^Zr]-ST-HDL mimicking NPs through the new approach for treating atherosclerosis in the future, the authors mentioned that the animal group size in the current study was limited for conducting statistical analysis of crucial individual markers [27].

## Conclusion and future perspectives

In this review, we summarized the recent progress of the development of HDL mimicking NPs for atherosclerosis treatment and imaging. The therapeutics encapsulated in HDL mimicking NPs were primarily chemical-based molecules, such as statin-based drugs. Only very few studies evaluated the synergetic effect of biomolecules agents and HDL mimicking NPs for atherosclerosis treatment. Therefore, in the future, it will be intriguing to study more about the delivery of biomolecule-based agents, such as novel RNAs, proteins, and DNAs, using HDL mimicking NPs.

Moreover, most studies shown here investigated the prevention of atherosclerosis progression by administrating HDL mimicking NPs with therapeutics in mouse models only at the early stage of atherosclerosis. Only few studies investigated the effect of therapeutic loaded HDL mimicking NPs in existing, advanced atherosclerotic plaque. Thus, future studies can consider the evaluation of these HDL mimicking NPs in large animal models that can form advanced atherosclerotic plaques.

In addition, as demonstrated in the review, large efforts were focused on the use of HDL mimicking NPs as therapeutic delivery systems for improving the therapeutic efficacy for atherosclerosis, while very few recent studies investigated the use of HDL mimicking NPs as imaging contrast and theranostic agents for atherosclerosis. Thus, future studies could concentrate more on those areas to better establish the overall functionality of these HDL mimicking NPs.

Lastly, the majority of the studies investigated the efficacy of HDL mimicking NPs using in vitro two-dimensional (2D) models, and ApoE^−/−^ mouse and rabbit models. However, in vitro 2D and in vivo models have their inherent limitations and cannot fully mimic the atherosclerosis pathology in clinical patients [65, 66]. Thus, future studies may also emphasize on the development of 3D in vitro atherosclerosis models to use along with in vivo models to provide a better evaluation approach to predict the efficacy and safety of these HDL mimicking NPs with therapeutic agents in humans.

## Data Availability

The datasets used and/or analyzed during the current study are available from the corresponding author on reasonable request.

## Abbreviations

HDL: high-density lipoproteins
NPs: nanoparticles
CVD: cardiovascular diseases
apoA-I: apolipoprotein A1
ABCA1: ATP-binding cassette transporters A1
SR-BI: scavenger receptor class B
RCT: reverse cholesterol transport
NO: nitric oxide
eNOS: endothelial NO synthase
rHDL: reconstituted high-density lipoprotein
rHDL NPs: reconstituted high-density lipoprotein mimicking NPs
PLGA: poly(lactic-*co*-glycolic acid)
ST-rHDL NPs: statin loaded reconstituted high-density lipoprotein mimicking nanoparticles
LT: lovastatin
HA: hyaluronic acid
HA-LT-rHDL NPs: hyaluronic acid modified lovastatin loaded reconstituted high-density lipoproteins mimicking nanoparticles
LT-rHDL NPs: lovastatin loaded reconstituted high-density lipoproteins mimicking nanoparticles
d-rHDL NPs: discoidal reconstituted high-density lipoproteins mimicking nanoparticles
s-rHDL NPs: spherical reconstituted high-density lipoproteins mimicking nanoparticles
AT: atorvastatin
GM1: monosialoganglioside
LOX-1: lectin-like oxidized low-density lipoprotein (LDL) receptor-1
HA-DOPE: hyaluronic acid-1,2-dioleoyl-sn-glycero-3-phosphoethanolamine
ECs: endothelial cells
ATP: adenosine triphosphate
miR-155: microRNA155
PL4: azide-phospholipid conjugate
9-DNA-PL4: azide-phospholipid conjugate core scaffold lined with 9-mer dsDNA
18-DNA-PL4: azide-phospholipid conjugate core scaffold lined with 18-mer dsDNA
POPC: 1-palmitoyl-2-oleoyl-sn-glycero-3-phosphocholine
DPPNOTE: *S*-nitrosylated phospholipid
SNO HDL mimicking NPs: *S*-nitrosylated phospholipid high-density lipoproteins mimicking nanoparticles
hAECs: human aortic endothelial cells
hAoSMCs: human aortic smooth muscle cells
CD40: cluster of differentiation 40
Gd: gadolinium
QD: quantum dots
PEG: polyethylene glycol
miR-210: microRNA-210
TPP: triphenyl phosphonium
GBCA-HDL: high-density lipoproteins as gadolinium based contrast agent
HDL-MNS: high-density lipoprotein-like magnetic nanostructures
LXR: liver X receptor
siRNA: small interfering ribonucleic acid
LOX-1 siRNA: lectin-like ox-LDL receptor-1 small interfering ribonucleic acid
Gd-DTPA-DMPE: gadolinium diethylenetriaminepentaacetic acid-1,2-dimyristoyl-sn-glycero-3-phosphoethanolamine
H4: amphipathic helices 4
H6: amphipathic helices 6
NER: normalized enhancement ratio
